# Trust in pharmaceuticals and vaccine hesitancy: exploring factors influencing COVID-19 immunization among Lebanese children aged 1 to 11 years

**DOI:** 10.1186/s12887-023-04394-3

**Published:** 2023-11-16

**Authors:** Emilie Feghaly, Venise Hanna, Nagham Karh Mohamad, Mohamad Ali Assaf, Juny Sebastian, Sami El Khatib, Rita Karam, Abeer Zeitoun, Atika Berry, Hajar Samaha, Marwa Shouman, Sahar Obeid, Souheil Hallit, Diana Malaeb

**Affiliations:** 1 School of Pharmacy, Lebanese International University, Bekaa, Lebanon; 2 College of Pharmacy, Gulf Medical University, Ajman, United Arab Emirates; 3 Department of Biomedical Sciences, Lebanese International University, Bekaa, Lebanon; 4 Center for Applied Mathematics and Bioinformatics (CAMB), Gulf University for Science and Technology, Mubarak Al-Abdullah, Kuwait; 5 Quality Assurance of Pharmaceutical Products Department, National Pharmacovigilance Program, Lebanese Ministry of Public Health, Beirut, Lebanon; 6 Department of Chemistry and Biochemistry, Faculty of Sciences, Lebanese University, Beirut, Lebanon; 7 Pharmacology Department, Faculty of Medical Sciences, Lebanese University, Beirut, Lebanon; 8 Preventive Medicine Department, Ministry of Public Health, Beirut, Lebanon; 9 Social and Education Sciences Department, School of Arts and Sciences, Lebanese American University, Jbeil, Lebanon; 10 School of Medicine and Medical Sciences, Holy Spirit University of Kaslik, P.O. Box 446, Jounieh, Lebanon; 11 Research Department, Psychiatric Hospital of the Cross, Jal Eddib, Lebanon; 12 Applied Science Research Center, Applied Science Private University, Amman, Jordan

**Keywords:** COVID-19 vaccine, Vaccine hesitancy, Knowledge, Children, Parents

## Abstract

**Introduction:**

The COVID-19 pandemic is a serious threat to everyone’s health. Numerous studies have demonstrated that vaccines are very effective in preventing COVID-19-related severe illness, hospitalization, and death. Children’s vaccination exerts its protecting effect by preventing the spread of the virus. The purpose of this study was to analyze the rate of COVID-19 immunization among Lebanese children aged 1 to 11 years and assess parental factors that affect immunization rates.

**Methods:**

An online cross-sectional study was conducted between January and March 2023. The online survey was distributed across all social media channels, including the Ministry of Public Health website.

**Results:**

A total of 390 parents filled the survey (mean age = 37.48 ± 8.39 years; 50.5% mothers; 70% with a university level of education). Mothers compared to fathers, having a history of bad reaction to a vaccine vs. not, and higher vaccine hesitancy were significantly associated with less willingness to administer the vaccine to the child. Trusting pharmaceutical companies was significantly associated with more willingness to administer the vaccine to the child.

**Conclusion:**

The results of this study show that the factors associated with parents’ decisions to vaccinate their children may vary. Our findings conclude that vaccine acceptance is being highly associated with parental concerns, trust, and information regarding the vaccine safety and efficacy.

**Supplementary Information:**

The online version contains supplementary material available at 10.1186/s12887-023-04394-3.

## Introduction

The introduction of COVID-19 vaccines had a significant impact on the global fight against the pandemic; multiple studies showed that vaccines were highly effective in preventing severe illness, hospitalization, and death caused by COVID-19 [1, 2]. According to the data reported from 164 countries, there was a significant decrease in the number of COVID-19 cases, hospitalization, and deaths [3]. In European Economic Area, the high vaccination rate was associated with a decrease in COVID-19 cases, and in both hospitalization and intensive care unit admissions among vaccinated individuals [4].

It had been discussed that higher levels of fear of being infected with COVID-19, the possibility of manifesting with severe symptoms, and the fear of the possible post-recovery complications after COVID-19 were associated with more willingness to get vaccinated [5]. The willingness of parents to vaccinate their children was highly influenced by the fear from COVID-19 vaccine [6]. Furthermore, vaccine hesitancy, possibly due to concerns regarding the safety of the vaccine or due to misconceptions regarding its potential efficacy, was an influential factor in decreasing the willingness to take the vaccine [7]. In addition to fear and hesitancy, parental knowledge about the vaccine affected its administration among children [8]. Specifically, the lack of knowledge regarding vaccine’s origin and effectiveness and its potential adverse events were associated with greater vaccine hesitancy [9]. In some countries such as China and Thailand, the refusal rate was reaching more than 50% [10, 11]. Several causes for parental refusal to vaccinate their children were reported; for instance, the majority (62.4%) of parents in Jordan and Iraq were not willing to vaccinate their children as they were concerned about adverse events and long-term problems because they believed that the vaccine was newly developed and lacked sufficient information about its effectiveness [12, 13]. The findings from parents from different countries with various characteristics imply that there were a lot of rumors and misconceptions regarding the COVID-19 vaccination [14]. In addition to hesitancy and fear that affected the willingness to administer COVID-19 vaccine to children, increased parental age, male gender, being married, having a higher level of education and higher income were associated with willingness to allow their children to receive the COVID-19 vaccine [15].

Lebanon started its COVID-19 mass vaccination campaign in February 2021, prioritizing vaccination mainly in healthcare workers, elderly, and individuals with underlying health conditions. As of April 28th, 2023, the total number of vaccine doses administered in Lebanon was 6,881,731 with 2,735,226 people fully vaccinated [16]. In September 2021, Lebanon started vaccinating children aged 12–17 years [17]. Vaccine hesitancy had been a concern in many Arab countries including Lebanon, with some individuals expressing concerns about the safety and efficacy of the COVID-19 vaccine, as well as mistrust in the government and healthcare system [18]. A survey conducted in Lebanon in August 2021 found that 68% of parents of children aged 12–15 years was willing to vaccinate their children against COVID-19, 14% were unsure, and 18% were not willing [19]. The main reasons for vaccine hesitancy included concerns about adverse events, lack of trust in the vaccine, and perceived low risk of COVID-19 in children [20, 21]. Thus, delving deeper into the possible causes of the parental decisions, is needed to assess the attitudes of Lebanese parents. Thus, the aim of this study was to examine COVID-19 vaccination rate among Lebanese children aged 1 to 11 years and investigate parental factors correlated with vaccination willingness in non-vaccinated children.

## Methods

### Study design

This was a cross-sectional study conducted from different governorates of Lebanon (Beirut, Beqaa, Mount Lebanon, South Lebanon, and North Lebanon) between January and March 2023, when vaccination was being actively administered to children. We used the Google Forms to collect the necessary data for the investigation where the project was advertised on social media and included an estimated duration. Indeed, participants were first invited to complete the questionnaire where a link was initially distributed via social media applications such as ‘WhatsApp’, ‘Instagram’ and ‘Facebook’, and then asked to share it with their acquaintances, friends and/or family members, explaining the snowball technique followed. The first page of the questionnaire included an explanation of the study topic, objectives, and a statement ensuring the anonymity of respondents. Parents with children aged between 1 and 11 years old and living in Lebanon were eligible to participate.

### Minimal sample size calculation

Based on a 32% reported rate of vaccinated children in a study conducted from the Eastern Mediterranean Region including Lebanon [18], the minimal sample size calculated according to the Epi Info software version 7.2 (population survey) was 335 participants to ensure a confidence level of 95%.

### Questionnaire

The questionnaire was self-administered in Arabic, the native language in Lebanon. It consisted of multiple sections:

### Sociodemographic data and general questions

This section of the questionnaire collected sociodemographic data of the participants, including age, gender, education level, monthly income, living region, marital status, and household crowding index calculated by dividing the number of persons by that of the rooms [22]. It also included questions about the COVID-19 infection history and its severity among the family member, child medical history, assessment of parents’ administration or willingness to administer the vaccine to children and previous adverse events from vaccine administration. Moreover, a question about the trust in the pharmaceutical companies for vaccine safety and effectiveness was also included.

#### Vaccine hesitancy questions

This section was developed from previous scales and published data about the aspects of vaccine hesitancy [19, 23]. It included six questions, assessed on a 5-point Likert scale from strongly agree (1) to strongly disagree (5). The score ranged between 6 and 30; the higher the total score, the higher the hesitancy towards the vaccine (Cronbach’s α = 0.73).

### Knowledge about COVID-19 scale

This scale included 18 questions that assessed knowledge and adapted from a previous study [19]. The questions and their right answer can be found in Appendix 1. The questions were assessed as yes, no, and I do not know. A scoring system was used by which a score of one is given to each correct answer and zero to each incorrect answer or I don’t know. The total score ranges from 0 to 18, with higher scores reflecting greater knowledge (Cronbach’s α = 0.68).

### Statistical analysis

Statistical Package for the Social Sciences (SPSS) 25 was used for the data analysis. Since the data was collected via a link, no missing values were recorded as all questions were required. The Chi-square test was used to compare categorical variables, whereas the Student t test was used to compare two means. Multinomial logistic regression was conducted, taking child vaccine administered/is willing versus did not/does not want to administer the vaccine as the dependent variable. All variables that showed a *p* < 0.25 were taken as independent variables in the final model. Significance was set at *p* < 0.05.

## Results

A total of 390 parents filled the survey (mean age = 37.48 ± 8.39 years; 70% with a university level of education and 50.5% were mothers). Our results showed that 44.9% admitted or are willing to administer the vaccine to their children. All other details about our sample are summarized in Table 1.

**Table 1:** Participants’ Characteristics (N = 390)

Parent gender	n (%)
Female	197 (50.5)
Male	193 (49.5)
**Child goes to school (yes)**	367 (94.1)
**Monthly income (in Lebanese Pounds)**	
< 3 million	86 (22.1)
3–10 million	107 (27.4)
> 10 million	197 (50.5)
**Marital Status**	
Married	375 (96.2)
Single	15 (3.8)
**Living region**	
City	241 (61.8)
Rural area	148 (37.9)
**Parent educational level**	
Secondary or less	117 (30.0)
University	273 (70.0)
**Family member infected with COVID-19 (yes)**	341 (87.4)
**Severe symptoms experienced were COVID-19 vaccine** ^**¥**^	
Yes	188 (55.1)
No	153 (44.9)
**Child infected with COVID-19**	
Yes	183 (46.9)
No	207 (53.1)
**Parents admitted or willing to administer vaccine to children**	
Yes	175 (44.9)
No	215 (55.13)
**Parent received vaccine (yes)**	330 (84.6)
**Experienced Adverse Events from vaccine (yes)**	286 (73.3)
**Child with chronic disease (yes)**	58 (14.9)
**Had bad reaction to vaccine**	146 (37.4)
**Trust pharmaceutical companies to deliver safe and effective vaccine**	177 (45.4)
**Parent age (years)**	37.48 ± 8.39
**Household crowding index**	0.32 ± 0.15
**Knowledge score**	13.18 ± 2.35
**Vaccine hesitancy scale score**	12.03 ± 5.08

Numbers are shown as n (%) or mean ± SD

¥ according to the number of family members infected with COVID-19

### Bivariate analysis

A significantly higher percentage of parents who administered or are willing to administer the vaccine to their children were fathers, have a child that goes to school, with a high monthly income, live in the city, had severe COVID-19 symptoms, had a child not yet infected with COVID-19, did not have a bad reaction to the COVID-19 vaccine, in those who trust pharmaceutical companies in making safe and effective vaccines, in those who received the COVID-19 vaccine, in those who experienced adverse events because of the vaccine and in parents who have a university level of education (Table 2). A lower mean parent’s age and vaccine hesitancy score were found in participants who administered/ are willing to administer the vaccine compared to not (Table 3).

**Table 2:** Bivariate analysis of categorical factors associated with child vaccine administration

Variable	Did not/does not want to administer the vaccine	Administered/ is willing to administer the vaccine	*P*
**Parent filling the survey**			**< 0.001**
Father	87 (45.1%)	106 (54.9%)	
Mother	128 (65.0%)	69 (35.0%)	
**Child goes to school**			**0.021**
No	18 (78.3%)	5 (21.7%)	
Yes	197 (53.7%)	170 (46.3%)	
**Monthly Income (Lebanese Pounds)**			**< 0.001**
< 3 million	55 (64.0%)	31 (36.0%)	
3–10 million	72 (67.3%)	35 (32.7%)	
> 10 million	88 (44.7%)	109 (55.3%)	
**Living Region**			**0.008**
City	120 (49.8%)	121 (50.2%)	
Rural	94 (63.5%)	54 (36.5%)	
**Family infected with COVID**			0.542
No	29 (59.2%)	20 (40.8%)	
Yes	186 (54.5%)	155 (45.5%)	
**Severe COVID-19 symptoms**			**< 0.001**
No	104 (68.0%)	49 (32.0%)	
Yes	82 (43.6%)	106 (56.4%)	
**Child infected with COVID-19**			**< 0.001**
No	93 (44.9%)	114 (55.1%)	
Yes	122 (66.7%)	61 (33.3%)	
**History of bad reaction to a vaccine**			**< 0.001**
No	68 (33.5%)	135 (66.5%)	
Do not know	28 (68.3%)	13 (31.7%)	
Yes	119 (81.5%)	27 (18.5%)	
**Trust pharmaceutical companies about safe and effective vaccine**			**< 0.001**
No	113 (86.3%)	18 (13.7%)	
Do not know	58 (70.7%)	24 (29.3%)	
Yes	44 (24.9%)	133 (75.1%)	
**Parent received the vaccine**			**< 0.001**
No	59 (98.3%)	1 (1.7%)	
Yes	156 (47.3%)	174 (52.7%)	
**Parent had adverse events from vaccine**			**< 0.001**
No	72 (69.2%)	32 (30.8%)	
Yes	143 (50.0%)	143 (50.0%)	
**Parent educational level**			**< 0.001**
Secondary or less	81 (69.2%)	36 (30.8%)	
University	134 (49.1%)	139 (50.9%)	
**Chronic diseases in child**			0.994
No	183 (55.1%)	149 (44.9%)	
Yes	32 (55.2%)	26 (44.8%)	

Numbers in bold indicate significant *p* values

**Table 3:** Bivariate analysis of continuous variables associated with child vaccine administration

Variable	Did not/does not want to administer the vaccine	Administered/ is willing to administer the vaccine	*p*	95% CI of the difference
Parent age	38.63 ± 9.09	36.08 ± 7.22	**0.002**	0.89; 4.21
Household crowding index	0.33 ± 0.16	0.32 ± 0.13	0.317	-0.01; 0.04
Knowledge	13.18 ± 2.39	13.18 ± 2.31	0.995	− 0.47; 0.47
Vaccine hesitancy	13.92 ± 5.89	9.72 ± 2.29	**< 0.001**	3.34; 5.06

Numbers in bold indicate significant *p* values

### Multivariable analysis

Mothers compared to fathers (aOR = 0.33), having a history of bad reaction to a vaccine vs. not (aOR = 0.25), and a higher vaccine hesitancy score (aOR = 0.86) were significantly associated with lower odds of willing to administer the vaccine to the child, whereas trusting pharmaceutical companies (aOR = 8.22) was significantly associated with higher odds of willing to administer the vaccine to the child (Table 4).

**Table 4:** Logistic regression taking child vaccine (administered/is willing versus did not/does not want to administer the vaccine*) as the dependent variable

Variable	aOR	*P*	95% Confidence Interval	
Person filling the survey (mothers vs. fathers*)	0.033	**0.002**	0.16	0.66
Child goes to school (yes versus no*)	2.21	0.250	0.57	8.57
Monthly income	0.503			
< 3 million Lebanese Pound	1			
3–10 million Lebanese Pound	1.02	0.960	0.42	2.52
> 10 million Lebanese Pound	1.52	0.365	0.62	3.73
Living region (rural versus city*)	0.87	0.678	0.44	1.71
Parent severe symptoms from vaccine administration (yes versus no*)	1.18	0.61	0.62	2.24
Child infected with COVID-19 infection (yes versus no*)	0.67	0.230	0.35	1.28
Bad reaction to a vaccine (yes versus no*)	0.25	**< 0.001**	0.13	0.49
Trust pharmaceutical company (yes versus no*)	8.22	**< 0.001**	3.69	18.31
Parent experienced adverse events from vaccine administration (yes versus no*)	1.34	0.436	0.64	2.82
Parent educational level (university vs. secondary or less*)	1.45	0.339	0.68	3.08
Parent age	0.99	0.743	0.95	1.04
Vaccination hesitancy	0.86	**< 0.001**	0.81	0.92

Nagelkerke R^2^ = 0.606. Numbers in bold indicate significant *p* values

## Discussion

The findings of this study shed light on the rate of COVID-19 immunization among Lebanese children aged 1 to 11 years and the parental factors that influence vaccine administration. The study revealed that being a mother, having experience a history of bad reaction to vaccine, and having higher vaccine hesitancy were associated with less willingness to vaccinate their children. However, having trust in the pharmaceutical companies was associated with greater willingness to administer the vaccine to the child. These findings suggest that parental attitudes and experiences play a significant role in the decision to vaccinate children against COVID-19.

Our findings showed that around 45% of the parents admitted or are willing to administer the vaccine to their children, which is higher than the data reported from a study conducted in the Eastern Mediterranean Region including Lebanon [18] probably because our study was conducted after the pandemic where several published data supports more vaccine safety and effectiveness compared to the reported data during the pandemic. The findings of this study related to the rate of children vaccination can be explained by the fact that the majority of the parents had higher educational level and socioeconomic status which is positively correlated with higher vaccination rate among children [24]. The possible explanation may be that parents with higher educational levels are more likely to have higher social status and therefore may have more channels to learn about the effects and adverse events of vaccines.

A high percentage of people who experienced severe symptoms following vaccine administration (55.1%) was found in our study, in opposite to the results of a national study in Lebanon that revealed the safety profile of the different COVID-19 vaccines administered during the Lebanese mass immunization campaign [25]. The high percentage of severe symptoms found in our study may be due to the convenient sampling technique followed to recruit participants.

This study showed that mothers are less willing to vaccinate their children compared to fathers which is in alignment with previous studies conducted in various countries, including Jordan [13], Iraq [12], Italy [26], Poland [27] and USA [28]. The findings of a multi-national study revealed that fathers were more receptive to vaccinating their children, particularly if they did not experience vaccine adverse events and had severe COVID-19 symptoms [18]. In fact, some mothers are responsible for health-care decisions concerning their children; they often prioritize their children’s well-being and may have doubts about the long-term effects of vaccines, especially new ones [13]. In addition, some mothers have concerns regarding the accurate and reliable information about COVID-19 vaccine safety, which can lead to hesitation and in turn may decrease the tendency to administer the vaccine to children [29].

This study showed that having bad experience with the vaccine manifested by adverse events was associated with less willingness to vaccinate the children, which is consistent with the results of a study conducted in Saudi Arabia [30]. According to a meta-analysis, exposure to COVID-19 vaccination and development of severe adverse events in parents raise concerns, increase hesitation, and decrease the willingness to administer the vaccine to their children [31]. Our results can be explained by the fact that personal experience to adverse events among parents trigger fear and worry that their children will experience similar adverse events or will have same adverse reactions if vaccinated [32].

Our study showed that having higher vaccine hesitancy was associated with less willingness to vaccinate children, in agreement with previous research conducted in different countries that have highlighted concerns and hesitancy among parents regarding the COVID-19 vaccine. Studies conducted in Jordan [13], Iraq [12], Italy [26] and Poland [27] have shown that parents express worries about vaccine safety, long-term effects, and the rapid development of the vaccine, which contribute to their lack of trust in the vaccine and its efficacy. These concerns are likely influenced by the spread of rumours and misconceptions about the vaccine.

In Lebanon, where the vaccination campaign commenced in February 2021, vaccine hesitancy has been a concern. A survey conducted in August 2021 found that 68% of parents of children aged 12–15 years were willing to vaccinate their children, while 18% were not willing [16]. The main reasons for vaccine hesitancy included concerns about adverse events, lack of trust in the vaccine, and the perceived low risk of COVID-19 in children. The current study expanded on these findings by examining the attitudes of parents for children aged 1 to 11 years, offering a more comprehensive understanding of the factors influencing vaccine administration.

The issue of trust in pharmaceutical companies has emerged as a significant factor contributing to willingness among parents to administer the COVID-19 vaccine to their children, in agreement with previous findings [33]. In terms of vaccination, public trust often refers to trust in the product itself, healthcare professionals, and policymakers [34]. Studies have highlighted the impact of mistrust on vaccine decision-making. Research conducted in different countries has consistently shown that individuals who express distrust in pharmaceutical companies are more likely to harbor concerns about vaccine safety and efficacy, leading to increased vaccine hesitancy [32]. To address this issue, it is crucial for public health authorities and healthcare professionals to acknowledge and understand parental concerns related to trust in pharmaceutical companies. Transparent communication, providing accurate information about the vaccine development process, regulatory oversight, and post-licensure monitoring can help build trust and alleviate concerns. Additionally, efforts should be made to ensure transparency in pharmaceutical company practices, foster collaboration between public health agencies and industry, and strengthen regulatory frameworks to enhance accountability and regain public confidence [35]. It is important to recognize that trust in pharmaceutical companies is a complex issue influenced by various socio-cultural, historical, and contextual factors. Tailoring interventions to address these concerns and engaging in ongoing dialogue with parents can contribute to improving vaccine confidence and uptake.

### Clinical implications

The study’s results have implications for public health interventions aimed at increasing COVID-19 vaccine uptake among children. Addressing parental concerns, providing accurate information about vaccine safety and efficacy, and building trust in the vaccine and healthcare system are crucial. Efforts should be made to counter misinformation and rumours, and healthcare providers’ involvement in vaccine recommendation and education can influence parental decision-making. Tailoring interventions and developing better communications for effective information dissemination to specific fathers and mothers, based on their unique concerns can also enhance vaccine administration.

### Limitations

It is important to acknowledge the limitations of this study. The cross-sectional design prevents establishing causal relationships, and self-reported measures may introduce response bias. The study sample was recruited through snowball sampling technique on social media platforms, potentially limiting its representativeness of the general population. Future research could address these limitations by utilizing larger and more diverse samples to further investigate factors influencing COVID-19 vaccine administration in children. Despite the rich demographic measures, we could have missed some subgroups of the population and some factors associated with the willingness to give the vaccine to the children aged between 1 and 11 years that could have changed our results, predisposing us to a residual confounding bias.

## Conclusion

This study provides insights into the rate of COVID-19 immunization among Lebanese children aged 1 to 11 years and the factors correlated with vaccine administration by parents. The results of this study suggest that factors associated with parental decisions to vaccinate their children may vary. Addressing parental concerns, building trust, and providing accurate information are crucial for increasing vaccine acceptance. Tailoring interventions to target specific parental groups can effectively address vaccine hesitancy. These efforts contribute to protecting children from COVID-19 virus and supporting global pandemic control. Future studies should be conducted to explore the rates of booster doses administration and assess the long-term safety and efficacy of COVID-19 vaccine.

### Electronic supplementary material

Below is the link to the electronic supplementary material.

Supplementary Material 1

## Data Availability

The datasets generated and/or analysed during the current study are not publicly available due to restrictions from the ethics committee but are available from the corresponding author on reasonable request.
