# Supplementary material for: Trust in pharmaceuticals and vaccine hesitancy: exploring factors influencing COVID-19 immunization among Lebanese children aged 1 to 11 years
Source: BMC Pediatr. 2023 Nov 16;23:570. doi: 10.1186/s12887-023-04394-3 (PMC10652549; doi:10.1186/s12887-023-04394-3)
Supplement: Supplementary file 1 — Supplementary Material 1 [file 12887_2023_4394_MOESM1_ESM.docx]

**Trust in Pharmaceuticals and Vaccine Hesitancy: Exploring Factors Influencing COVID-19 Immunization among Lebanese Children aged 1 to 11 years**

1. What is your gender?
   1. Male
   2. Female
2. Age:
3. Was is the parent marital status?
   1. Married
   2. Single
4. Are you currently living in?
   1. Rural area
   2. City
5. How many rooms are there in your home? (excluding kitchen and bathrooms)
6. How many people are living in the same home?
7. What is the parent educational level?
   1. Secondary or less
   2. University
8. Was is the parent monthly income (in Lebanese Pounds)?
   1. < 3 million
   2. 3-10 million
   3. >10 million
9. Was any of the family members infected with COVID-19?
   1. Yes
   2. No
10. Were the symptoms severe?
    1. Yes
    2. No
11. Did you take the vaccine?
    1. Yes
    2. No
12. Did you experience adverse events from the vaccine?
    1. Yes
    2. No
13. Do you trust the pharmaceutical companies to deliver safe and effective vaccine?
    1. Yes
    2. No
    3. Don’t know
14. Was the child infected with COVID-19?
    1. Yes
    2. No
15. Did you administer the vaccine to your child or willing to administer the vaccine to your child?
    1. Yes
    2. No
16. If yes, did your child have bad reaction to the vaccine?
    1. Yes
    2. No
17. Does your child have chronic diseases?
    1. Yes
    2. No

**Knowledge about COVID-19 Scale**

|  | **Yes** | **No** | **Do not Know** |
| --- | --- | --- | --- |
| Fever is a symptom of the Coronavirus | x |  |  |
| Cough is a symptom of the Coronavirus | x |  |  |
| Sore throat is a symptom of the Coronavirus | x |  |  |
| Body pain is a symptom of the Coronavirus | x |  |  |
| Diarrhea or constipation is a symptom of the Coronavirus | x |  |  |
| Headache is a symptom of the Coronavirus | x |  |  |
| When coronavirus infection is suspected, I will measure the fever first | x |  |  |
| When coronavirus infection is suspected, I will avoid unnecessary daily activities | x |  |  |
| To avoid infection, I avoid contact with individuals suspected of being infected with the Coronavirus | x |  |  |
| Washing hands with soap and water can remove the cause of the disease | x |  |  |
| The disease can be transmitted directly through coughing | x |  |  |
| The disease can be transmitted directly through contact with contaminated surfaces | x |  |  |
| The disease can be transmitted directly through consumption of contaminated milk and meat |  | x |  |
| The disease can be transmitted through contact with infected people (shaking hands, hugging, kissing) | x |  |  |
| The disease is more serious in pregnant women | x |  |  |
| The disease is more serious in the elderly | x |  |  |
| The disease is more serious in people with a weak immune system | x |  |  |
| The disease is more serious in people with cancer, diabetes, chronic respiratory diseases, and hypertension/heart problems | x |  |  |

X represents the correct answer

**Vaccine Hesitancy Questions**

|  | Strongly Agree | Agree | Disagree | Strongly disagree |
| --- | --- | --- | --- | --- |
| Childhood vaccines are effective |  |  |  | x |
| Having my child vaccinated is important for his/her health |  |  |  | x |
| All childhood vaccines offered by the government program in my community are beneficial |  |  |  | x |
| The information I receive about vaccines from the vaccine program is reliable and trustworthy |  |  |  | x |
| Generally, I do what my doctor or health care provider recommends about vaccines |  |  |  | x |
| I am concerned about dangers of vaccines | x |  |  |  |
